# Ileal benign tumor (Vanek’s tumor) a rare cause of bowel obstruction in elderly adult: a case report

**DOI:** 10.1093/omcr/omaf043

**Published:** 2025-08-11

**Authors:** Daniel Lopez-Zertuche, Roberto Garcia-Manzano, Carlos A Zacaula-Aguilar, David A Peña-Di-Donato, Guillermo Espinoza Zarazúa, Erika M Hernandez-Montiel

**Affiliations:** Department of Surgery, Dr. Manuel Gea González General Hospital, Mexico City 14080, Mexico; Department of Surgery, Dr. Manuel Gea González General Hospital, Mexico City 14080, Mexico; Department of Surgery, Dr. Manuel Gea González General Hospital, Mexico City 14080, Mexico; Department of Pathology, Dr. Manuel Gea González General Hospital, Mexico City 14080, Mexico; Department of Pathology, Dr. Manuel Gea González General Hospital, Mexico City 14080, Mexico; Department of Surgery, Dr. Manuel Gea González General Hospital, Mexico City 14080, Mexico

**Keywords:** intussusception, Vanek’s tumor, small bowel obstruction, Ileal neoplasms, case report

## Abstract

Inflammatory fibroid polyps (IFP) are rare benign lesions of the gastrointestinal tract that can lead to serious complications, such as intussusception, particularly in adults. We present a case of small bowel intussusception caused by IFP, emphasizing diagnostic and therapeutic challenges. The patient presented with acute abdominal pain and signs of intestinal obstruction, confirmed by computed tomography, with the tumor identified during transoperatory and histopathological examinations confirmed the diagnosis of IFP. This case highlights the importance of considering IFP as a differential diagnosis in adult bowel obstruction secondary to intussusception, and the need for timely surgical intervention to prevent complications. The literature contains some case reports of IFPs that explaining the management and diagnosis of this pathology. Our objective was to communicate our experience of IFP as the etiology of intestinal obstruction in an elderly adult.

## Introduction

Inflammatory fibroid polyps (IFPs), also known as Vanek’s tumors, are rare benign submucosal lesions of the gastrointestinal (GI) tract that were first described by Vanek in 1949 [1]. Most commonly located in the stomach and small intestine but may occur throughout the GI tract [2–5]. Histologically, IFPs arise from the submucosal layer and are characterized by spindle-shaped cells, vascular and fibroblast proliferation, and infiltration of inflammatory cells, predominantly eosinophils [1–3]. Immunohistochemical staining has revealed positive expression of specific markers, which may be linked to their etiopathogenesis, although the exact mechanisms remain unclear. Potential contributing factors include trauma, allergic reactions, genetic predispositions, and other unidentified stimuli [2, 4]^.^

Clinical complications, including intussusception, a rare condition in adults that accounts for approximately 1% of intestinal obstructions [1–3]. The clinical presentation may vary, including abdominal pain, nausea, vomiting, and other signs of obstruction [3–5]. Imaging modalities, particularly computed tomography (CT), are crucial for diagnosing adult intussusception [4]. Surgical resection remains the treatment of choice for resolving the obstruction and removing the underlying lesion [2–4, 6].

This article presents a case of small bowel intussusception caused by an IFP with a focus on the clinical presentation, diagnostic approach, and surgical management of this rare entity.

## Case report

A 73-year-old female Latin American patient had a history of an uncomplicated right hip replacement. The patient presented with abdominal pain that started 10 days earlier. Initially, the pain was described as mild and colicky, localized to the mid-abdomen, accompanied by nausea. The patient sought consultation with an external physician who diagnosed her with irritable bowel syndrome and prescribed antispasmodics and antiemetics for 5 days. Despite this treatment, the patient’s condition progressively worsened, with the onset of abdominal distension, persistent nausea, fecal vomiting, and a 24-h history of absent bowel movements and flatulence.

Upon presentation to the emergency department, physical examination revealed tachycardia, significant abdominal distension, palpation exacerbated the pain in the mid-lower abdomen without palpable tumor. Rectal examination revealed an empty ampulla with no evidence of bleeding or palpable tumor, and a preserved sphincter tone.

**Figure 1:**
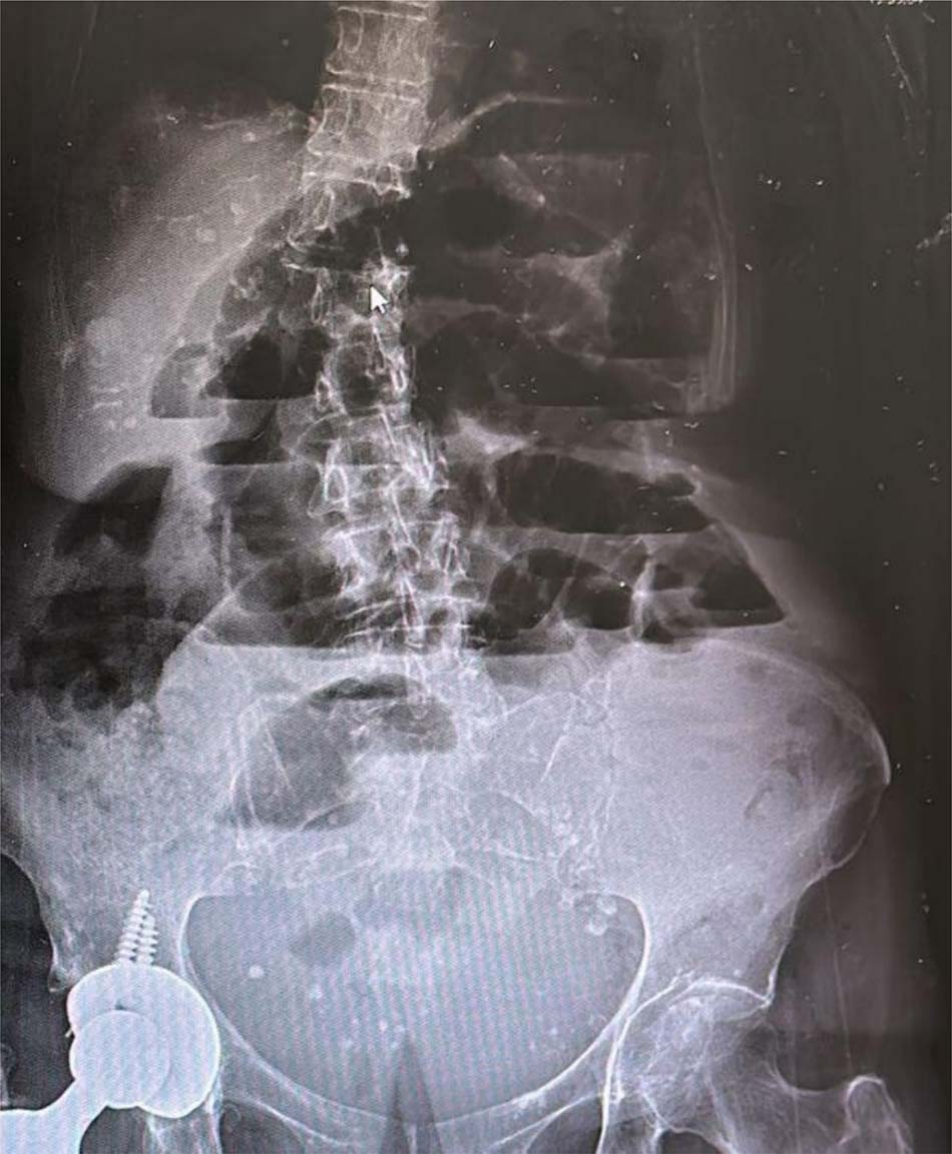
Abdominal radiography with air-fluid levels. Dilation of intestinal loops with multiple air-fluid levels.

**Figure 2:**
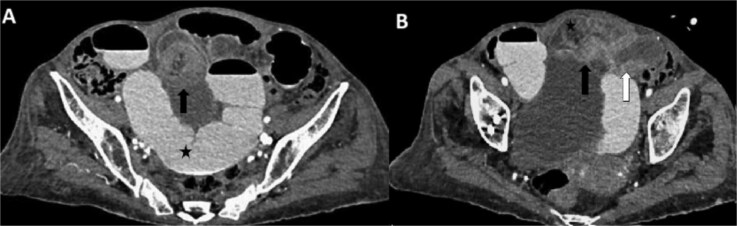
Abdominal CT scan with oral and intravenous contrast with intussusception features in axial view: (A). There is generalized dilation of the small intestine (black star) with contrast medium present within the lumen. At the ileal level, a rounded, heterogeneous tubular structure is noted, dependent on the loops of the small intestine, exhibiting a ‘target sign’ (black arrow) alongside increased wall thickness and edema. (B). Protrusion through another rounded mass, creating a ‘bowel-within-bowel’ appearance, characterized by an amorphous lesion acting as a lead point (black arrow) arising from the distal ileum (white arrow), which is causing intussusception in the proximal ileum (black star).

Laboratory results within the reference values. Abdominal radiography revealed air-fluid levels (Fig. 1). The double-contrast abdominal CT revealed intussusception in the distal ileum (Fig. 2).

**Figure 3:**
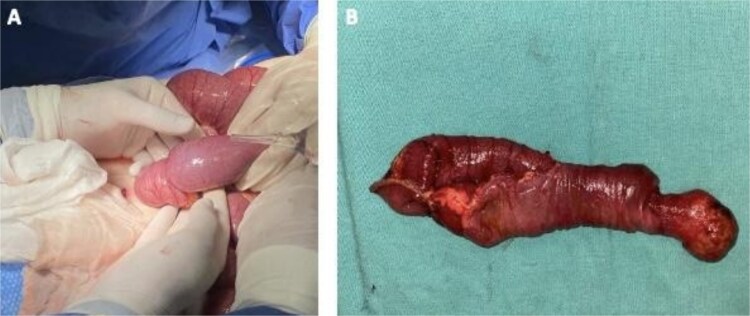
Ileo-ileal intussusception and resected segment with polyp. (A) Ileum-ileal intussusception segment. (B) 15 cm resection of ileum with the appearance of a polyp of 3.5x2.5 cm.

**Figure 4:**
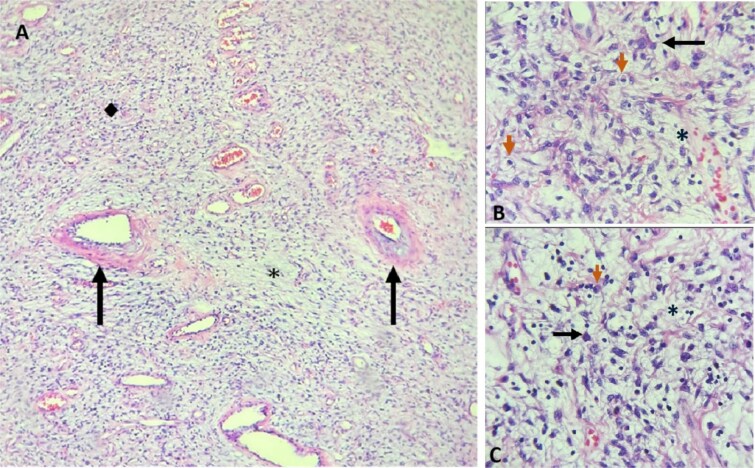
Microscopic features of IFP. A: H&E, 10x: Hypercellular (◆) and hypocellular (*) areas are observed. The hypocellular areas show a myxoid matrix. The blood vessels are thin-walled, with perivascular ‘onion-skinning’ thickening (black arrows), accompanied by lymphocytes, plasma cells, and eosinophils. B and C: H&E, 20x: Hypercellular and hypocellular areas are observed. The hypocellular areas show a myxoid matrix (*). Star-shaped cells (black arrows) are observed, as well as fusiform cells, accompanied by lymphocytes, plasma cells, and eosinophils (orange arrows).

Preoperative management included intravenous resuscitation, nasogastric tube insertion, and antibiotic therapy with ceftriaxone and metronidazole. Diagnostic laparoscopy was performed by Hasson technique for safer approach, revealing segmental dilation of the intestine and allowing adequate surgical exposure. A 15 cm ileo-ileal intussusception was identified 105 cm proximal to the ileocecal valve. However, after 30 minutes, the procedure was converted to exploratory laparotomy due to carbon dioxide retention, hemodynamic instability, and difficulty reducing the intussusception laparoscopically. Manual reduction attempts were unsuccessful, opting for intestinal resection followed by side-to-side enteroenteric mechanical anastomosis. The resected specimen revealed a 3.5 cm stenotic polyp (Fig. 3). Histopathological analysis revealed proliferation of spindle cells, ‘onion-skinning’ pattern, and stroma infiltration by inflammatory cells (Fig. 4).

She was discharged on postoperative day 6 following an uneventful recovery. A follow-up assessment conducted two weeks later revealed a continued uncomplicated recovery, with no signs of any postoperative complications.

## Discussion

Inflammatory fibroid polyps (IFPs) are rare benign lesions of the GI tract that can lead to significant complications, including intussusception and bowel obstruction, particularly in adults. Although IFPs account for only about 1% of adult bowel obstructions, they are an important differential diagnosis due to their potential to mimic malignant conditions [1, 3]. IFPs have a slight predilection for females, with a female-to-male ratio of 1.3:1, and most commonly present in the fifth decade of life [4]. The majority of IFPs are located in the stomach (66%–75%), followed by the small intestine (18%–21%), predominantly in the ileum, and less frequently in the colon (4%–8%); involvement of other regions of the gastrointestinal tract is rare, occurring in less than 3% of cases [2–5].

Histopathologically, IFPs are characterized by spindle cell proliferation and eosinophilic infiltration [2, 4]. Immunohistochemically, they typically exhibit positive staining for CD34 and vimentin, negative staining for CD117, and overexpression of PDGFRA [2–4]. Diagnosis is typically based on the distinctive morphological characteristics of the lesion [4]. IFPs show low Ki67 expression (<1%), a feature that helps distinguish them from malignant tumors, particularly gastrointestinal stromal tumors (GIST), which may share similar location, histology, and some immunohistochemical markers [2].

IFPs can present with a wide range of symptoms, from asymptomatic cases to acute intestinal obstruction, often due to the tumor acting as a lead point for intussusception [2, 6, 7]. Computed tomography (CT) imaging is crucial for diagnosing intussusception, even when the lead point is not immediately visible [6, 8].

Although IFPs can be removed endoscopically, depending on their location, surgical intervention remains the standard and most effective treatment for adult intussusception. Resection with primary anastomosis is typically curative [2, 4, 9]. Small intestine polyps carry a higher risk of intussusception, requiring prompt surgery [2, 8]. Key factors like the mass’s location, size, viability of the invaginated segment, and risks of seeding or venous dissemination during manipulation should be carefully considered, especially when preoperative histopathological diagnosis is not available. In such cases, resection without reduction is recommended [2, 3, 8]. Exploratory laparoscopy or laparotomy are considered the first choices for resolution [3, 7]. Postoperative complications in cases of intestinal resection and anastomosis are directly related to their integrity and functionality, such as dehiscence, stenosis, and anastomotic fistulas.

The prognosis for patients with resected IFPs is excellent, with rare or no reported recurrence or metastasis [4]. Due to their benign nature, routine follow-up is generally unnecessary. However, histopathological examination is essential to rule out other causes, including malignancies. The most common benign gastrointestinal lesions are adenomatous polyps, typically small in size. Intestinal lipomas contain fat, which is visible on CT and MRI. Lymphomas, accounting for 20% to 40% of malignant small bowel lesions, are usually large endoluminal tumors. GISTs, which are related to IFPs, often display partial extraluminal growth, irregular margins, and a heterogeneous appearance. GISTs are most commonly found in the stomach as polyp-like growths, able to cause intussusception, but immunohistochemistry differentiate from IFPs showing positivity for CD34 and CD117 [2, 10].

This case underscores the importance of considering IFPs in the differential diagnosis of adult patients with small bowel obstruction representing a surgical emergency in adults [7, 8]. Early diagnosis and timely surgical intervention are critical for preventing complications and ensuring favorable outcomes.

## Sources of funding

The Article Processing Charge (APC) was covered by the Universidad Nacional Autónoma de México (UNAM) through a Read & Publish agreement. The authors received no other financial support for the research, authorship, and publication of this article.

## Patient Consent

Not required in view of the case report and anonymous information, and the collected data without any patient identifiable data.

## Guarantor

Daniel Lopez Zertuche.
